# Investigating tooth morbidity risks, prevalence, and interventions in tribal setting: a study protocol focused on the Irula community in Tamil Nadu

**DOI:** 10.3389/fdmed.2024.1427597

**Published:** 2024-09-10

**Authors:** Margret Beaula Alocious Sukumar, Roshni Mary Peter, Alex Joseph

**Affiliations:** ^1^ SRM School of Public Health, SRM Institute of Science and Technology, Chennai, India; ^2^ Department of Community Medicine, SRM Medical College Hospital and Research Centre, SRM Institute of Science and Technology, Chennai, India; ^3^ Division of Epidemiology, SRM School of Public Health, SRM Institute of Science and Technology, Chennai, India

**Keywords:** caries, periodontitis, quality of life, tooth morbidity, elderly, tribal

## Abstract

**Aim:**

Dentistry is uniquely positioned as a healthcare profession, distinguished from allied health or paramedical fields. It operates within a university-based structure, maintaining primary care responsibilities. Oral diseases impose a substantial worldwide health and economic burden, profoundly affecting the well-being of those affected. This cross-sectional study is centered on the Irula tribes in Tamil Nadu, India. Investigating the extent of tooth morbidity and loss, identifying possible risk factors, looking at oral hygiene habits, and evaluating the oral health-associated quality of life (OHRQoL) in this population are the main goals. As this population is at high risk for dental issues, promoting good oral hygiene becomes crucial. Indigenous populations, including the Irula tribes, have been underrepresented in research studies. The vulnerability of the Irula tribes is exacerbated by their remote locations, resulting in limited access to fundamental healthcare facilities.

**Materials and methods:**

This study employs a cluster sampling approach, aiming to include 880 individuals aged 60 and above from Kancheepuram and Chengalpattu districts. The methodology encompasses a community-based questionnaire, clinical assessments utilizing standardized indices, and the assessment of the Oral Health-Related Quality of Life (OHRQoL). The Statistical Package for Social Services, version 20, will be used to analyze all of the data that has been gathered (SPSS).Categorical variables will undergo analysis, with descriptive statistics and frequency percentages computed.

**Conclusion:**

This study evaluates tooth morbidity and dentistry's role in screening chronic diseases like diabetes among the Irula tribes. It explores risk factors, oral hygiene practices, treatment seeking behavior, and oral health-related quality of life to gain a comprehensive understanding.

## Introduction

Dentistry demands a comprehensive understanding of basic clinical medicine to address the needs of individuals facing chronic conditions, which compromise their biological and pharmacological states. Dental professionals are increasingly involved in primary care activities, such as screening for diabetes and managing hypertension, highlighting the integral role of dentistry in overall health (1).

Oral diseases stand among the most prevalent health issues worldwide, imposing significant burdens on both health and economy while diminishing affected individuals' quality of life. With over 3.5 billion cases globally, many oral diseases are preventable, yet their risk and severity are exacerbated by the rising prevalence of chronic conditions, particularly among older adults (2). Tooth morbidity encompasses various clinically observable abnormalities, including decay, dental caries, and periodontal disease, with global prevalence reaching 45% over the last three decades. Caries and periodontitis are primary contributors to tooth loss, which not only incurs significant rehabilitation costs but also profoundly affects overall well-being (3–5). Tribal communities represent a notable indigenous segment, comprising 9.01% of India's total population, primarily concentrated in the central and western regions. Often residing in remote, mountainous areas with limited access to technology, education, and economic opportunities, tribal communities experience significant marginalization. In Tamil Nadu alone, 794,697 individuals belong to Scheduled Tribes (ST), with 36 distinct groups identified. Among them, the Kattunayakan, Kotas, Irulas, Paniyas, Kurumbas, and Todas are designated as particularly vulnerable tribal groups (PVTG) by the Indian government (6).

The Irula community, deriving its name from the Tamil word “Irul” meaning darkness, resides predominantly in districts like Tiruvallur, Kancheepuram, and Tiruvannamalai. With occupations mainly centered around snake and rat trapping, the Irula people possess extensive knowledge of herbal resources and vegetation (7).

Studies among the Irula tribe indicate a high prevalence of chronic illnesses and poor oral health status, yet literature on their oral health remains scarce compared to other tribal populations (8–10). Given the significant disease burden faced by the Irula community, prioritizing oral health research becomes imperative to devise effective preventive measures and address disparities in oral health outcomes.

Therefore, urgent assessment of dental morbidity within the Irula population is essential to inform and shape effective health policies tailored to their needs.

### Study objectives

1. To assess the prevalence of tooth morbidity and tooth loss among the Irula tribes of Tamil Nadu.
2. To identify caries, non caries lesions and periodontitis related risk factors among the tribes.
3. To explore their oral hygiene practices and dental treatment-seeking behaviour of the Irula tribes.
4. To assess the explanatory capacity of leading psychological theories, create interventions based on these theories, and measure their effectiveness in enhancing the oral health of elderly individuals.

### Hypothesis

Null Hypothesis (H0): The prevalence of tooth morbidity among the Irula tribal population in Tamil Nadu is not significantly different.

Alternative Hypothesis (H1): There is a significant difference in the prevalence of tooth morbidity among the Irula tribal population in Tamil Nadu.

## Materials & methods

The research duration spanned from January 2023 to February 2024. Ethical clearance was obtained from the Institutional Ethics Committee at the School of Public Health, SRMIST (IEC Protocol Number 0039/IEC/2023), ensuring adherence to ethical standards throughout the study process. The team visited the Tribal Welfare Department in Chepauk, Chennai, to gather information regarding the hamlets and population lists of the Irula Tribes in Tamil Nadu. Later, they sought permission to initiate the project in the Irula districts of Tamil Nadu.

The study employed a cross-sectional design among the Irula tribes of Tamil Nadu. Cluster sampling was utilized to gather the required sample from Thiruvallur districts. Using a prevalence rate of 50.1% from a study by Shah et al (11), the sample size was calculated with a confidence interval of 98%, a 5% error term, a design effect of 2%, and a 10% non-response rate, resulting in an overall sample of 880 individuals. With an extensive review of literature, discussions with professional experts, and reference to scientific papers, a semi-structured questionnaire was designed to assess the prevalence of tooth morbidity.

The study tools aimed to assess the prevalence of tooth morbidity among the Irula tribe. Section A dealt with demographic variables such as age, gender, occupation, education, and income. Section B addressed information on medical history, personal habits, and dietary practices, comprising 20 questions. Section C covered information on oral health practices, quality of life, and examination of the oral cavity, comprising 72 questions. The content validity of the tool was established based on the opinion of dentists, epidemiologists, and public health experts of SRM SPH. Minor suggestions in the structure of the questionnaire, such as changes in the opinion of items and alterations in questions, were incorporated into the tools and finalized for the pilot study. The questionnaire was converted into a digital format and uploaded onto portable computing devices.

The study employed various instruments, including curved probes, straight probes, mouth mirrors, WHO probes (CPITN), cotton rolls, chip blowers, and explorers for dental examination purposes. Focus group discussions involving 8−10 stakeholders were conducted following a standardized plan and script. Post-session, transcripts and notes were generated, and a summary of the discussions was compiled. The entire process was audio-recorded, translated into English, documented, and subjected to analysis.

Tooth morbidity, encompassing carious lesions, non-carious lesions, and periodontitis, was assessed using the International Caries Detection and Assessment System (ICDAS) (12–14) for caries detection, the Smith and Knight tooth wear index for non-carious lesions, and the Community Periodontal Index for Treatment Needs (CPITN) index for periodontal disease (15). Tooth mobility assessment adhered to Miller's classification. Data collection was conducted by the validated questionnaire from the pilot survey and qualitative observations. Furthermore, quality of life assessment was carried out utilizing the Geriatric Oral Health Assessment Index (GOHAI) (16). The participants were screened for examination of oral cavity by one and the same dentist. The instruments used were sterilized using a standard protocol (17).

### Assessment of theoretical frameworks

To gauge the effectiveness of theoretical models in understanding oral health behaviors, participants will undergo structured interviews during their initial assessment. This process will entail gathering data on various sociodemographic factors such as gender, age, education level, occupation, and housing type, alongside oral health behaviors including dietary habits, toothbrushing routines, and interdental cleaning practices.

The questionnaire will encompass scales corresponding to three prominent psychological theories:

a. Health Belief Model: This model posits that health-promoting behaviors are influenced by six key domains, including perceived susceptibility, severity, barriers, benefits, cues to action, and self-efficacy.
b. Theory of Planned Behavior: Suggests that behavioral intentions and actions are influenced by intention, subjective norms, and perceived behavioral control.
c. Social Cognitive Theory: It underscores that behavior is shaped by individuals' outcome expectations, self-efficacy, and beliefs regarding the effectiveness of actions in achieving desired outcomes.

### Development of theory-driven intervention

Theoretical frameworks demonstrating the highest explanatory power concerning oral health behaviors will guide the development of targeted interventions. In instances where no single model exhibits sufficient explanatory capability or when the most suitable model varies across behaviors, consideration will be given to employing two models simultaneously with the highest fit. An expert panel comprised of a behavioral scientist, health psychologist, public health researcher, and dental specialist will collaborate to craft theory-driven interventions. These interventions will be meticulously designed to address all constructs and domains within the selected theory or model, with careful consideration given to the needs, interests, and health literacy of the target demographic, particularly older adults from diverse social and educational backgrounds.

### Pilot testing and randomization

A small-scale pilot study involving 15–20 older adults will be conducted to assess the relevance, feasibility, and acceptability of the developed interventions. Subsequently, participants will be randomly allocated into two intervention groups (1:1 ratio), stratified by gender and educational level. Randomization will be conducted by a researcher not involved in data collection, utilizing computer-generated allocation sequences that remain undisclosed until interventions are assigned.

### Intervention strategies

Participants in the two intervention groups will receive either conventional health education or theory-driven interventions:

Conventional Health Education: Participants will receive oral health pamphlets covering topics such as common oral diseases, dental problems among older adults, the significance of oral self-care practices, and proper techniques for tooth brushing and interdental cleaning.

Theory-Driven Intervention: This intervention will utilize multimedia materials (e.g., printed materials, PowerPoint presentations, short videos, tooth models) and group activities (e.g., quizzes, discussions, demonstrations, practice sessions, feedback) tailored to address all constructs and domains of the selected theory or model. Additionally, participants will receive three rounds of mobile phone messages aimed at providing ongoing support and facilitating behavioral changes.

### Statistical analysis plan

The data recorded underwent analysis using the Statistical Package for the Social Sciences, SPSS Statistics for Windows, Version 23.0 (IBM Corp., Armonk, NY). Descriptive statistics were conducted to determine percentages, means, and standard deviations. For inferential statistics, a Chi-square test, independent *t*-test, and one-way analysis of variance were performed. The comparison of means will be conducted using either an independent samples *t*-test. A significance level of *p*-value ≤ 0.05 was established.

## Discussion

The health-related attitudes, actions, and cultural norms individuals adhere to greatly influence their well-being. Racial and ethnic perspectives and values can either promote or deter individuals from accessing healthcare services, as evidenced by research indicating that economically disadvantaged groups and ethnic minorities exhibit lower tendencies to seek medical assistance. India harbors the second-largest elderly population globally, trailing only China, with around 80 million individuals. This demographic cohort is expanding at a more rapid rate compared to the overall population.

Health challenges in tribal communities are profoundly influenced by factors such as social, cultural, educational, economic, and political practices. The limited access to basic healthcare facilities renders tribal populations highly susceptible to diseases (18, 19).

Among the approximately 40 tribal communities in Tamil Nadu, the Irulas stand out as particularly distinctive. They possess extensive knowledge of medicinal plants, especially in treating snake bites and various ailments (20). The epidemiological literature on oral health in the elderly, especially in tribal populations, paints a less optimistic picture, indicating significant disparities among countries and regions, further compounded by institutionalizations resulting from aging populations. In the future, dental practitioners will face the challenge of caring for an unprecedented number of older adults. Particularly in developed countries, these older adults will increasingly demand a higher volume of dental services (19).

Dental caries, often regarded as a consequence of affluence and modernization, pose a substantial risk to geographically isolated and traditionally primitive populations. Limited access to modern dental practices, coupled with passive oral healthcare, insufficient utilization, low awareness levels, economic constraints, and high rates of illiteracy, contribute significantly to the heightened vulnerability of such populations to dental caries (21).

This study is unique first of its kind as this study identifies all tooth morbidity conditions among the older adults of Irula tribes and its risk factors. This study also highlights their treatment seeking behaviour and their perceptions towards oral health. This study does not pertain to prevalence; instead, our focus is on raising awareness regarding stigma, advancements in policy, and fostering extensive collaborations with a renewed commitment to dispel misconceptions about oral health among the older adults.

Given that dietary intake, tooth brushing, and flossing are the targeted behaviors, it's worth noting that both diet and personal hygiene are shared risk factors associated with both oral health and systemic conditions. Consequently, the intervention holds relevance for enhancing the overall health of the elderly population. Moreover, oral health is inseparable from general health, making an intervention geared towards improving oral health among older adults likely to enhance their nutritional status, daily functionality, and overall quality of life.

The study underscores the significant oral health challenges faced by tribes in the Thiruvallur district of Tamil Nadu. It highlights the urgent need for implementing a comprehensive oral health program tailored to the needs of this population. Such a program should encompass free emergency dental care and basic preventive treatments, offered at affordable rates and delivered by well-trained tribal primary oral health care providers.

To ensure the program's effectiveness, it must penetrate deep into tribal settlements, with active involvement from local individuals educated in medicine and tribal leaders. Moreover, addressing cultural and traditional customs that contribute to poor oral health is paramount. By focusing on transforming unhealthy practices while preserving beneficial ones, these programs can instigate positive changes and improve the oral health outcomes of tribal communities.

## Conclusion

This study aims to assess the prevalence of tooth-related morbidity and explore dentistry's potential for screening chronic diseases like diabetes. It investigates risk factors, oral hygiene practices, dental treatment seeking behavior, and oral health-related quality of life among the older adults of Irula tribes, offering a comprehensive understanding of their oral health status. Establishing dedicated oral health policies is crucial given the significant social and economic impacts of these conditions. There's a pressing need to enhance and expand oral health programs to cater to the millions requiring dental services nationwide.
